# Shell Growth of Large Benthic Foraminifera under Heavy Metals Pollution: Implications for Geochemical Monitoring of Coastal Environments

**DOI:** 10.3390/ijerph17103741

**Published:** 2020-05-25

**Authors:** Nir Ben-Eliahu, Barak Herut, Eyal Rahav, Sigal Abramovich

**Affiliations:** 1 Department of Earth and Environmental Science, Ben-Gurion University of the Negev, Beer Sheva 84105, Israel; nirbene@post.bgu.ac.il; 2 National Institute of Oceanography, Israel Oceanographic and Limnological Research, Haifa 31080, Israel; barak@ocean.org.il (B.H.); eyal.rahav@ocean.org.il (E.R.)

**Keywords:** larger benthic foraminifera, heavy metals, pollution, physiological resilience, symbionts, marine ecology, coastal monitoring, sclerochronology

## Abstract

This study was promoted by the recent efforts using larger benthic foraminiferal (LBF) shells geochemistry for the monitoring of heavy metals (HMs) pollution in the marine environment. The shell itself acts as a recorder of the ambient water chemistry in low to extreme HMs-polluted environments, allowing the monitoring of recent-past pollution events. This concept, known as sclerochronology, requires the addition of new parts (i.e., new shell) even in extreme pollution events. We evaluated the physiological resilience of three LBF species with different shell types and symbionts to enriched concentrations of Cd, Cu, and Pb at levels several folds higher than the ecological criteria maximum concentration (CMC) (165–166, 33–43, 1001–1206 µg L^−1^, respectively), which is derived from aquatic organisms’ toxicity tests. The physiological response of the holobiont was expressed by growth rates quantified by the addition of new chambers (new shell parts), and by the chlorophyll *a* of the algal symbionts. The growth rate decrease varied between 0% and 30% compared to the unamended control for all HMs tested, whereas the algal symbionts exhibited a general non-fatal but significant response to Pb and Cu. Our results highlight that shell growth inhibition of LBF is predicted in extreme concentrations of 57 × CMC of Cu and 523 × CMC of Cd, providing a proof of concept for shell geochemistry monitoring, which is currently not used in the regulatory sectors.

## 1. Introduction

Monitoring of heavy metals (HMs) in the marine environment is traditionally done by combining analyses of water, sediments, and tissues of the local biota. These compartments, especially the water-phase components (dissolved and particulate), provide a snapshot of the specific time of sampling and therefore require long-term recording [1,2]. Each method has its pros and cons; water analysis provides direct measurements of pollutant levels, but it is prone to overlooking events of temporally confined pollutant effluents since it is limited by the frequency of sampling. The analysis of pollutants in the sediment substrate indicates time-averaged concentrations that reflect sedimentary processes and sediment properties rather than short- to medium-term pollution load [1]. No method indicates the impact of the toxicity (i.e., biological response) of the pollution on the ecosystems [3]. The analysis of tissues has the advantage of recording the presence of biologically available metals, as well as their effect and possible toxicity on the organisms [3]. However, different organisms exposed to the same conditions differ in the accumulation of various metal concentrations with variation in the biological response (i.e., vital effects related to metabolism). Thus, the need for a long-term recording (i.e., bio-archive) is not resolved [2]. To overcome these disadvantages, chemical analysis of continuously accreting biomineralized structures (known as sclerochronology) was suggested as a bio-archive to document temporal trends in chemical pollution [2].

One of the most suitable calcifying organisms for sclerochronology is benthic foraminifera, based on the efficiency of these protozoa in recording water chemistry and its physiological impact continuously during the growth of their carbonate shells [4,5]. Foraminifera are unicellular marine organisms with short reproductive cycles and fast-growing rates [6]. They are easily collected from sediment or rocks with high abundances in small samples [7], providing a large database for statistical analysis. Living benthic foraminifera is now considered as one of the most important indicators of the environment status of any marine habitat mainly based on field population studies [7,8,9,10,11,12,13,14,15,16,17,18,19]. This study was promoted by the recent efforts of using benthic foraminiferal shells’ geochemistry as reliable and accessible living data loggers for HMs in seawater. Several laboratory experiments and field studies were set to find the HMs’ threshold concentrations and their effect on the foraminifera growth rates and population response [20,21,22,23,24,25,26,27,28], pseudopodia activity [22,24,29], symbionts response [30], cytological and morphological alterations [20,25,28,29,30,31,32,33,34,35,36] (i.e., their vitality), as well as the partition coefficients of HMs between the foraminifera shell and the ambient seawater [4,21,37,38]. Previous studies have examined the physiological response of different benthic foraminifera species to very extreme conditions of 10–500 µg L^−1^ (120 days) [31] and 3–11 µg L^−1^ Cu (82 days) [21], and 0–20 mg L^−1^ Cd (30 days) [22] that are orders of magnitude higher than its ‘typical’ ambient concentrations.

The goal of this study was to test the applicability of larger benthic foraminifera (LBF) for geochemical monitoring of HMs pollution in shallow marine environments (coastal habitats). The studied species were selected based on their cosmopolitan nature and dominant presence in most tropical shallow water and reef environments. Specifically, our study aimed to experimentally evaluate the ability of *Amphistegina lobifera*, *Amphistegina lessonii*, and *Sorites orbiculus*, to produce shells (new chambers) under extreme concentrations of Cd, Cu, and Pb (by a factor of 4–9 to the acute ecological criteria). We also tested the resilience of their endosymbionts considering their potential impact on their host physiology, including calcification processes. Considering the experimental efforts, we selected three HMs that are anthropogenically introduced into the coastal waters via point and non-point land-based sources and are known as toxic pollutants in the marine environment, including in the eastern Mediterranean sea coastal water.

## 2. Materials and Methods

### 2.1. Selected Species

The studied species were chosen to represent the two main groups of symbionts bearing LBF with different biomineralization mechanisms (i.e., porcelaneous vs. hyaline) and endosymbionts (dinoflagellates vs. diatoms): *A. lobifera* and *A. lessonii* (hyaline shell with diatoms as endosymbionts) and *S. orbiculus* (porcelaneous shell with dinoflagellates). Samples containing *A. lobifera* and *S. orbiculus* were collected in October 2018 from an abrasion rock platform in the Mediterranean Sea near Tel-Shikmona (Lat. 32.825368 N, Lon. 34.954788 E, Israel), at 0.2–0.5 m water depths. Specimens of *A. lessonii* were collected in March 2019 from rock pebbles in the Gulf of Aqaba-Eilat, Red Sea (Lat. 29.502205, N Lon. 34.918023 E, Israel), at water depths of 1–2 m. Both locations are considered as relatively clean environments and thus do not involve a pre-exposure acclimation of the local foraminifera to high HMs contaminators [39,40].

### 2.2. Experimental Culturing with HMs Additions

The study was based on a manipulative culturing system exposing living specimens of the studied species to ~4–9 × CMC (criteria maximum concentration) of Cd, Cu, and Pb (Table 1) and one control treatment with ambient seawater collected from Tel Shikmona (Lat. 32.825368 N, Lon. 34.954788 E, Israel). These high concentrations were chosen to represent extreme pollution scenarios in coastal environments (i.e., higher HMs concentrations than their CMC, Table 1) based on the United States Environmental Protection Agency (EPA) National Recommended Water Quality Criteria-Aquatic Life Criteria Table (https://www.epa.gov/wqc/national-recommended-water-quality-criteria-aquatic-life-criteria-table), and to test whether LBF are still able to calcify under these expected stress conditions. Each treatment consisted of three biological replicates. Each replicate of *A. lobifera* and *A. lessonii* consisted of 30 specimens (90 specimens per treatment), and for *S. orbiculus*, 17 specimens per replica were collected (51 specimens per treatment). The culture vessels were placed under 25 °C, 45 PAR (photosynthetically active radiation, µmol photons m^−2^ s^−1^) and 12:12 light and dark cycles in a temperature-controlled climate chamber for 24 (*A. lessonii*), 31 (*A. lobifera*), and 32 (*S. orbiculus*) days. The solutions were refreshed every week to maintain stable HMs concentrations and pH and salinity conditions. Measurements included a fresh solution after 1 week of culturing. The ecological CMC (synonymous with “acute”) is a regulatory value of the aquatic life criterion under the ambient water quality criteria (AWQC). It represents the highest level for a 1-h average exposure of a chemical in the water that aquatic organisms can be exposed without resulting in an unacceptable effect, calculated based on toxicity tests. The AWQC CMC address aquatic organisms’ toxicity, and ensures that HMs levels are below levels considered harmful to the development of aquatic communities. The CMC values of the studied HMs in marine waters are summarized in Table 1 according to the screening quick reference tables (SQuiRTs) of National Oceanic and Atmospheric Administration (NOAA) [41,42]. This study referred to the CMC that were set for Cd, Cu, and Pb as a base unit, allowing normalization between the HMs concentrations (e.g., 1 × CMC, 2 × CMC etc.). The CMC or acute concentration values are updated with time and differ between countries and their regulators. For example, the CMC of Cd was updated in 2016 from 40 µg L^−1^ to 33 µg L^−1^ from its latest update in 2001 [42], different values from the Australian and New Zealand guidelines, which stands at 5.5 µg L^−1^ [43], or the Canadian guidelines (CWQG), which recommend a chronic value of 0.12 µg L^−1^ and provides no recommended value for acute exposure [44]. This study was not aimed for a particular criterion of the acute concentration but rather to test high environmental levels. Solution preparation and validation are presented in Supplementary Material 1.

In the current study, the physiological state was quantified by counting new chambers produced during the exposure to HMs as a proof of concept for sclerochronology, considering the potential use of foraminifera as a tool for single chamber analyses, which relies on the addition of new parts [4]. The identification of new chambers was based on the fluorescent calcite marker approach. Before the beginning of the experiments, the specimens were kept in ambient seawater and spiked with 20 µM of the green calcein probe (sigma) [45]. The specimens were re-examined after 7 days for new chambers labeled in green fluorescent color and only specimens that added at least one new chamber (as in alive and active) were chosen for the experiments. The labeled chambers were used to distinguish between new chambers produced during the experiments without the calcein probe. Examining of the specimens was done using an epifluorescence stereomicroscope (Leica M165 FC) and a digital microscope color camera (Leica MC120 HD) combined with white light (ring illuminator, Leica LED5000 RL) and fluorescence illuminator led light (Leica SFL100, 530 nm, Pmax 10W) sources. A filter set optimum was used for the detection of emitted green and red colors (ET-GFP-LP, excitation ET480/40x, emission ET510-LP). Growth of *S. orbiculus* was also calculated based on the size measurement of differences in the shell’s area between the beginning and the end of the experiment, due to the species’ discoidal shell shape and annular subdivided chambers. The size measurements were done using ImageJ (v1.46) software [46] based on digital images with a scale bar as a landmark.

The physiological responses of the algal symbionts were quantified by in vitro measurements of the chlorophyll *a* (Chl *a*) concentrations in randomly selected single specimens using a Chl *a* non-acidification fluorescent module in a Turner Designs Trilogy Laboratory Fluorimeter (Trilogy, Turner designs) following EPA Method 445.0 [47]. Specimens were randomly chosen for each treatment (12 specimens for *A. lobifera* and *S. orbiculus*, 18 specimens for *A. lessonii*) and the Chl *a* was extracted by adding 1.5 mL of Acetone 90% [48]. The Chl *a* content was normalized to the area of the specimens. The data analysis is based on images of the specimens and their shell size measured using ImageJ (v1.46) software [46]. The effects of HMs pollution on the algal symbionts were also measured in vivo by measuring the Chl *a* fluorescence [3,49,50,51]. An image analyses approach, which compares the red channel’s digital numbers (DNs) of Chl *a*-stimulated fluorescence images, was used on all studied living specimens of *A. lessonii*. This approach is based on the positive correlation between the Chl *a* concentration to the intensity of the red channel (I_red_) of fluorescence images [52]. The I_red_ is calculated by dividing the DN with the exposure time of the image. Dead specimens were determined by the loss of symbiont coloring and were excluded from the image analyses. To clean unwanted DN from the analyses, extreme low (0–0.125, dark gray-black) and high (0.825–1, light gray white) DNs were excluded for representing the black background and white scale bar. The fluorescence images were taken under dark conditions with an exposure time of 450–500 ms, 0.55–0.6 gamma, and 1–1.2 gain settings. Except for the imaging time, the specimens were maintained in cold and dark conditions inside the freezer (−18 °C) or inside a cooler box filled with ice packs when moved out of the freezer. Survival rates were calculated in all treatments based on the number of living and dead specimens. Specimens that underwent reproduction events were considered live and were recognized by their unique shell breakage.

### 2.3. Culturing Conditions Monitoring

The pH and salinity influence the physiological state of marine organisms and foraminifera, and therefore, both were monitored during the experiments for sustaining the natural ambient levels [53,54,55,56]. Measurements of both pH and salinity were made using the Multi 3320–Meter for 2 Sensors (WTW Xylem Analytics, Weilheim in Oberbayern, Germany). The pH of the solutions was measured using a SenTix 41 (WTW Xylem Analytics, Weilheim in Oberbayern, Germany) electrode and the salinity was measured using a TetraCon 325 electrode (WTW Xylem Analytics, Weilheim in Oberbayern, Germany).

### 2.4. Statistical Analysis

All statistics were performed using R software version 3.5.3 (the R Foundation, Vienna, Austria). Most of the data were found to have a non-normal distribution according to the Shapiro–Wilk test, so the Mann–Whitney nonparametric test was used between the control and HMs treatments. Significant differences were accepted at *p* < 0.05. The data is presented in the results chapter as mean values (of the treatments and the control) and their standard deviations. Data and statistics results are included in the Supplementary Material 2.

## 3. Results

### 3.1. Foraminiferal Growth

**Figure 1:**
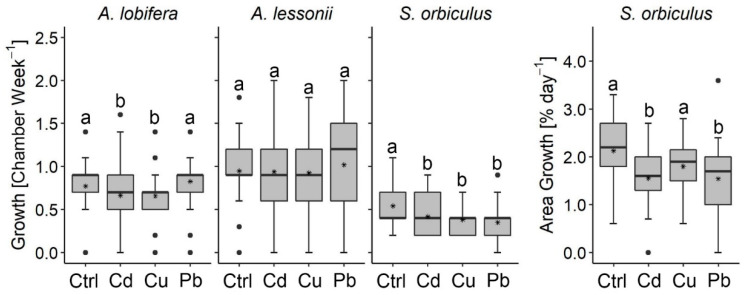
Growth rates of the studied species. Letters (a, b) represent significant differences between the control and the heavy metals treatments (Mann–Whitney nonparametric test, *p* < 0.05). Note: the significantly lower growth rates of all HMs-treated specimens of *Sorites orbiculus* and the Cd- and Cu-treated specimens of *Amphistegina lobifera.* The number of specimens used to calculate the rates in every boxplot is ca. 90 in the *Amphistegina* species and ca. 51 in *S. orbiculus*. Errors are the standard deviation of three replicates. Data and statistics results are included in the Supplementary Material 2.

**Figure 2:**
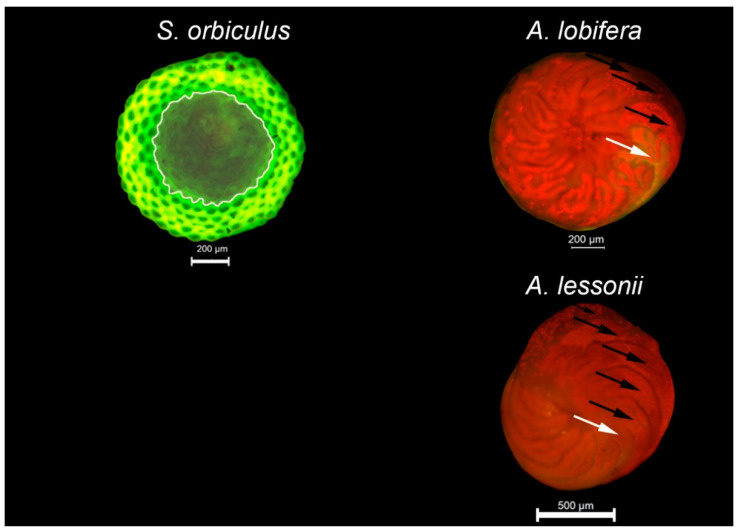
Shell growth observations based on the fluorescent calcite marker approach. For *Amphistegina lobifera* and *Amphistegina lessonii*, the existing green fluorescence-labeled chambers (white arrows) were used to distinguish from new chambers produced during the experiments without the calcein probe (black arrows). For *Sorites orbiculus*, the existing shell area produced before the experiments without the calcein probe (area inside the white line) was distinguished from the new shell area produced during the experiments with the calcein probe (area outside the white line).

All treatments resulted in high survival rates (above 90%), and the data is included in the Supplementary Material 2. All three studied LBF species produced new chambers in all treatments, indicating clear growth and thus a non-fatal response (Figure 1). However, a minor negative response was observed in some of the treatments: *A. lobifera* recorded a significant decrease in the number of new chambers between the control (3.6 ± 1.1) and the Cu and Cd treatments (3.0 ± 1.0 and 3.0 ± 1.4, respectively) but not between the control and the Pb treatment (3.6 ± 1.2). *Sorites orbiculus* showed a significant negative response of growth in all HMs treatments (2.0 ± 0.9, 1.8 ± 0.7, 1.7 ± 0.8 for Cd, Cu, and Pb, respectively) compared to the control (2.5 ± 0.8) based on the decreased number of new chambers. Significant area addition in *S. orbiculus* was found between the control and the Cd and Cu treatments. In contrast, no significant differences in the growth rates were found in *A. lessonii* between the HMs treatments (3.1 ± 1.6, 3.1 ± 1.6, 3.4 ± 1.7 for Cd, Cu, and Pb, respectively) and the control (3.2 ± 1.4), yet the specimen’s growth rate variances increased between the control and the HMs treatments. Interestingly, the mean growth rate of the Pb-treated *A. lobifera* and *A. lessonii* was non-significantly higher than the control specimens. Figure 2 illustrates the final shell growth observations and identifications of new chambers and shell area.

### 3.2. Algal Symbionts

**Figure 3:**
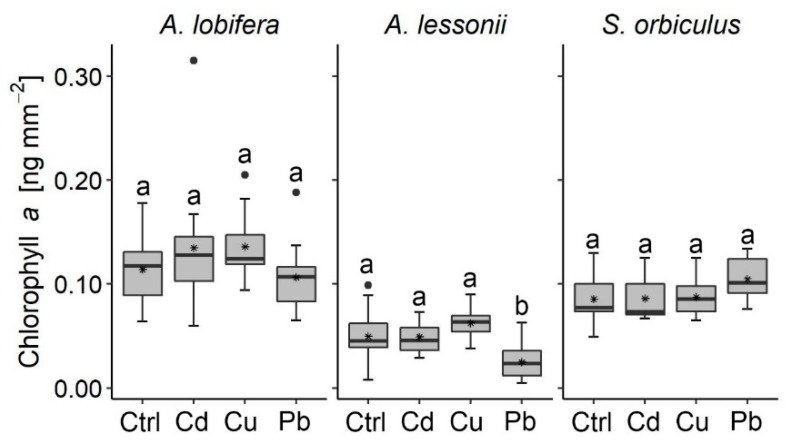
In vitro chlorophyll *a* concentrations of the studied species. Letters (a, b) represent significant differences between the control and the heavy metals treatments (Mann–Whitney nonparametric test, *p* < 0.05). Boxplots comprise of 12 specimens in each treatment for *Amphistegina lobifera* and *Sorites orbiculus*; and 18 specimens for *Amphistegina lessonii*. Note: the decrease of Chl *a* in the Pb treatment of *A. lessonii*. Error is the standard deviation. Data and statistics results are included in the Supplementary Material 2.

In general, most of the algal symbionts of the studied species did not record a negative response to the HMs treatments as indicated by their similar Chl *a* concentration compared to the control (Figure 3). The only exception is found in the Pb treatment of *A. lessonii*, which recorded a decrease in the Chl *a* concentration (0.11 ± 0.02 ng mm^−2^).

**Figure 4:**
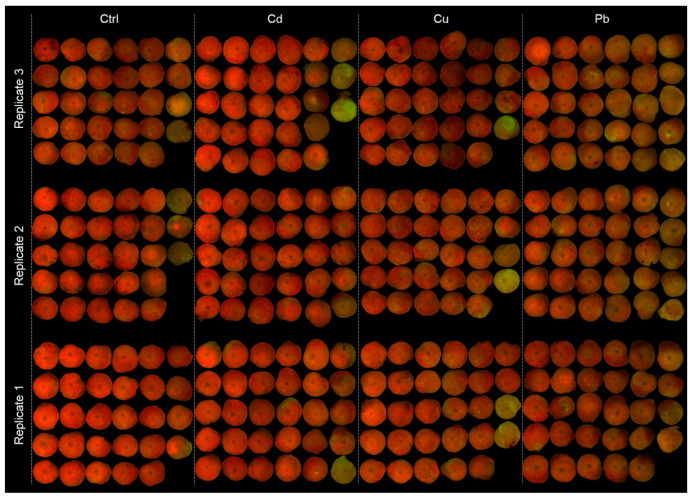
Fluorescence images of all living cultured specimens of *Amphistegina lessonii*. The red colors are formed by stimulating the fluorescence of chlorophyll *a*. Note: the vivid red colors of the specimens from the control and Cd treatment and the faded red colors of specimens from the Pb and Cu treatments. The image’s size was scaled for the collage presentation; the average diameter is 1.4 ± 0.5 mm.

A semi-quantitative proxy of Chl *a*-stimulated fluorescence images was used to further test the negative algal response to the HMs treatments in *A. lessonii* (Figure 4). The collage images of the cultured specimens show vivid red colors of the specimens in the control and Cd-enriched treatment and common faded red and green colors in specimens of the Pb and Cu treatments, indicating a decrease in the photo-active Chl *a* pigments in the latter.

**Figure 5:**
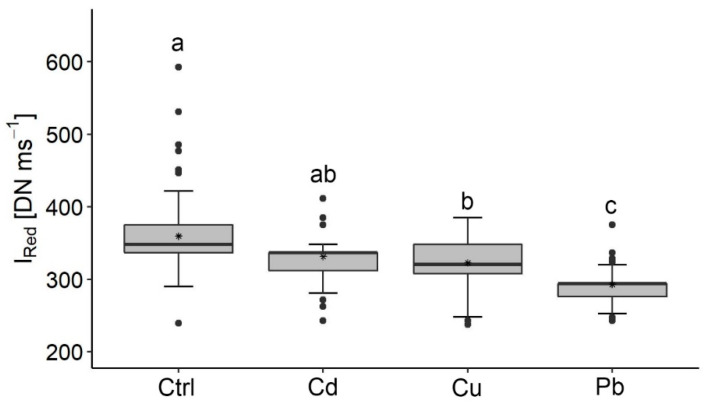
I_red_ values quantified from the fluorescence images. Letters (a, ab, b, c) represent significant differences between the control and heavy metals treatments. Note: the higher I_red_ values of the control compared to the Cu and Pb treatments and the higher I_red_ value of Cd and Cu treatments compared to the Pb treatment. Number of images processed in each treatment: Control, N = 81; Cadmium, N = 82; Copper, N = 82; Lead, N = 84. Error is the standard deviation. Data and statistics results are included in the Supplementary Material 2.

These observations were quantified by the I_red_ analyses, which indicated significant differences between the control and the Pb and Cu treatments (Figure 5). The highest values of I_red_ are found in the control while the lowest values are found in the Pb treatment.

### 3.3. Culturing Conditions Results

The monitoring results of pH and salinity are included in the Supplementary Material 1. The lowest pH values measured during the experiments were 7.8, 7.9, 7.9, 7.6 (*A. lobifera*), 8.0, 7.8, 8.0, and 7.9 (*A. lessonii*), and 8.0, 8.0, 8.0, and 7.9 (*S. orbiculus*), for the control, Cd, Cu, and Pb treatments accordingly. Among these results, a pH value of 7.6, found in the Pb treatment of *A. lobifera*, is known to reduce the growth rates of several benthic foraminifera species [53,55,56], yet the growth rate results in this study show that the Pb-treated *A. lobifera* specimens had similar growth rates to the control (Figure 1). The salinity was stable in all treatments and replicates of *A. lessonii*, ranging between 38 and 39 ppt, values that are not known to cause negative effects on the growth of foraminifera.

## 4. Discussion

### 4.1. LBF Shell Growth under HMs Extreme Pollution

Calcification processes are often used as an indicator of the well-being of foraminifera [20,22,24,34,48,57,58,59,60,61,62,63,64]. We hypothesized that the studied LBF will negatively respond to the extreme HMs concentration culturing treatments by reducing shell growth rates (or even reach shell growth inhibition) and lowering algal symbionts of Chl *a* concentrations. Our results show that the shell growth of the studied LBF was only mildly or not affected by the high HMs concentrations, indicating minor stress on calcification processes. This is particularly true for the *Amphistegina* species, which were less effected compared to *S. orbiculus*.

**Figure 6:**
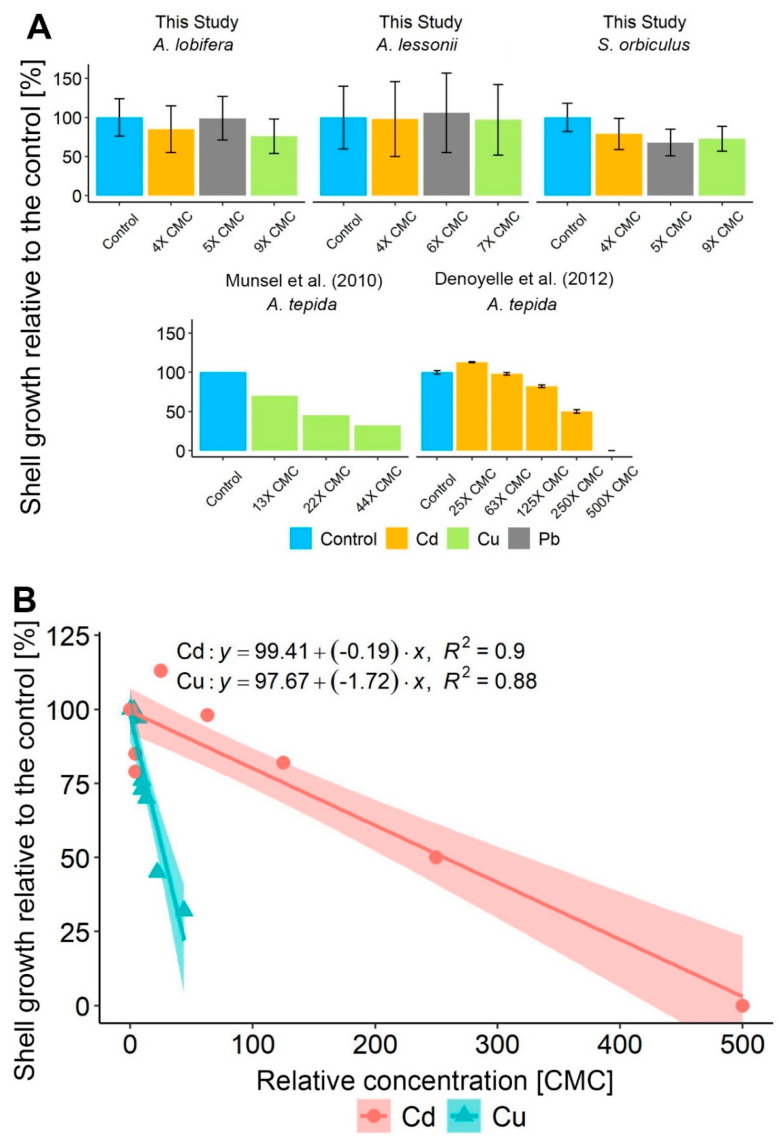
(**A**) Relative growth rates to the control between different benthic foraminifera species of the current study and modified results from previous studies with *Ammonia tepida* [21,22] when exposed to additions of Cd, Cu, and Pb. Error in the current study is the standard deviation of three replicates. (**B**) Comparison between relative growth rates to the control of different benthic foraminifera species of the current study and modified results from previous studies with *Ammonia tepida* [21,22] between exposure to additions of Cd and Cu. Note: the significant sharp decrease of the relative growth rates in the Cu additions compared to Cd additions (two-way ANOVA, F value = 18, *p* < 0.05). Data and statistics results are included in the Supplementary Material 2.

An exceptional renowned small benthic foraminifera species is *Ammonia tepida*, which can survive high HMs concentrations [21,22,29]. Comparisons of our results to previous similar studies with *A. tepida* [21,22] (Cd 0–20 mg L^−1^, 30 days; Cu 3–11 µg L^−1^, 82 days) indicate that the studied LBF are highly resilient when chronically (24–32 days) exposed to Cd, Cu, and Pb additions, showing zero to a maximum 30% decrease in growth at 4–9 × CMC (Figure 6a). This observation is also supported by the Cd and Cu linear model’s coefficient of determination (Cd R^2^ = 0.9, Cu R^2^ = 0.88) of the relative growth rates in additions of 0–43 × CMC Cu and 0–500 × CMC Cd (Figure 6b) in the current and previous studies [21,22]. Among these HMs, Cu was found to negatively affect foraminiferal growth 9 times more profoundly than Cd, expressed by the significant steeper linear regression slope of Cu (Figure 6b, Two-way ANOVA, F value = 18, *p* < 0.05). This result was expected by the lower Cu concentration, which was set as CMC compared to Cd (Table 1).

Additional comparisons further highlight the relatively high resilience of LBF concerning shell growth compared to most other benthic foraminifera. For example, *Pseudotriloculina rotunda* and *Rosalina leei* (Table 2) sharply reduced their growth rate when exposed to additions of 1 × CMC and 0.1 × CMC of Zn (70 days) and Hg accordingly [20,24] (66 days) and *Pararotalia nipponica* reduced the maximum growth in 0.1 × CMC of Cd (21 days) [34]. Our results demonstrate the advantage of LBF as recorders of HMs pollution by their exceptional growth rates, up to 5 times higher than smaller benthic foraminifera (Figure 1, Table 2). These observations do not rule out the existence of other impacts on cellular processes due to HMs toxicity. For example, it has been shown recently a reduced pseudopodial activity and enhanced intracellular lipid droplets under Cd-treated specimens of *Ammonia parkinsoniana* [29].

The tolerance and growth response of each of the studied species to additions of Cd, Cu, and Pb might be species specific or, in a broader concept, point to tolerance differences between species of different shell structures. This notion is observed by the non-significant growth decrease of *S. orbiculus* (porcelaneous shell structure) compared to the *Amphistegina* species (hyaline shell structure) (Figure 6a). Several laboratory studies have shown a connection between HMs exposure to morphological deformities [20,21,23,32,33]. Additionally, several field studies suggested that miliolid species (porcelaneous shell structure) are more easily affected by environmental stresses, which results in deformed shells [65,66]. While no obvious deformations were found in *P. rotunda* (porcelaneous shell) when exposed to Zn, SEM images revealed an anomalous orientation of calcite crystals [24]. Deformation was also absent in *A. tepida* and *Heterostegina depressa* (hyaline shell structure) when exposed to Cu [37]. Several studies show that foraminifera may have different detoxification mechanisms to cope with HMs that suggest an induced oxidative stress, including thickening of the inner organic lining, an increase in the amount and size of lipid droplets, degenerated mitochondria, residual bodies’ proliferation, and oxidative stress biomarkers [30,31,32,36]. These cytological alterations may be exhibited without morphological deformations [31,32,35] and present a physiological response of the foraminifera when the shell growth rate does not vary. In the current study, no deformed shells were observed, although new chambers were produced. Nevertheless, cytological alterations were not studied and might have occurred.

### 4.2. Algal Symbionts Response to HMs Pollution

Chl *a* is commonly used as a proxy for algal symbionts’ biomass found in benthic organisms (e.g., corals; foraminifera) [48,67,68,69,70,71,72]. In this study, Chl *a* was used to evaluate the possible effects of HMs on the performance of the algal symbionts. In general, both types of algal symbionts exhibit a non-fatal response to all HMs treatments. Yet, variable responses of the symbiont algae were observed between the studied foraminifera species. The dinoflagellates within *S. orbiculus* and the diatoms within *A. lobifera* recorded similar biomass values between the HMs treatments and the control, indicating tolerance to the exposure levels of the HMs whilst the diatom assemblage within *A. lessonii* showed a negative response to the Pb treatment indicated by their reduced Chl *a* concentration and to both Pb and Cu by their I_red_ levels. The different algal symbionts’ sensitivity of the *Amphistegina* species to HMs found in this study may be attributed to differences in the diatom assemblages of the two species [57,64]. LBF may have an adaptation mechanism against environmental stressors that induces bleaching, helping the selection of more tolerant specimens [73,74,75]. For example, *Amphistegina* species may have the ability to acclimate to stressful conditions by its capacity to host a wide variety of diatoms [76] and select more stress-tolerant symbionts’ taxa [77]. Surprisingly, Cu, which is considered the most toxic HM to microalgae among those tested in this study [78,79], lowered the Chl *a* photo-active density less than Pb.

Another interesting observation is the discrepancy between the Chl *a* measurements and I_red_ analyses in recording the negative effect of Cu on the diatoms symbionts of *A. lessonii*. This discrepancy implies that the number of specimens used for the Chl *a* measurements was insufficient to record the decrease in Chl *a* due to the high variability between specimens. This observation highlights the advantage of image analyses in recording the physiological response of foraminiferal symbionts.

The negative response of the host and symbionts was not correlated, which points to a disassociation in their stress expressions to exposure to the Cd, Cu, and Pb additions. A similar lack of correlation was also observed by [80], who proposed that the confined position of the symbionts within the endoplasm limits their degradation effects to the inner cell, while the ectoplasm continues to feed and produce new chambers.

## 5. Conclusions

1. All studied LBF species showed high tolerance to chronic exposure of 4–9 × CMC of Cu, Cd, and Pb.
2. The studied LBF species showed higher shell growth rates than most smaller benthic foraminifera.
3. A minor but statistically significant decrease in shell growth was found in *S. orbiculus*, indicating moderate stress but continuous calcification.
4. Algal symbionts exhibited a general non-fatal response. The dinoflagellates symbionts within *S. orbiculus* and the diatoms symbionts within *A. lobifera* showed tolerance to the exposure of Cd, Cu, and Pb with no negative response detected, while the diatoms within *A. lessonii* negatively responded to the Pb and Cu treatments.
5. Pb was found to negatively affect the algal symbionts more than the foraminifera host and Cu was found to negatively affect both the foraminifera as a host and the algae symbionts, affecting the organism as a holobiont. Cu was found to negatively affect the growth more than Cd and Pb.
6. The continuous formation of the shell (new chambers) during exposure to extreme levels of HMs concentrations supports the applicability of LBF shells as living geochemical loggers of coastal pollution, a method currently not used in the regulatory sectors.

## Figures and Tables

**Table 1:** Concentrations (µg L^−1^) of Cd, Cu, and Pb tested in this study and their reference to ecological criteria. Errors represent the standard deviation of all the refreshments of the solutions, including measurements of a fresh solution a solution after 1 week of culturing. Criteria maximum concentration (CMC) values according to the screening quick reference tables (SQuiRTs) of the National Oceanic and Atmospheric Administration (NOAA) [41,42].

Heavy Metal	CMC	This Study	
		*A. Lobifera, S. Orbiculus*	*A. Lessonii*
Cadmium	33	166 ± 3 (4 × CMC)	165 ± 3 (4 × CMC)
Copper	4.8	43 ± 1 (9 × CMC)	33 ± 1 (7 × CMC)
Lead	210	1001 ± 41 (5 × CMC)	1206 ± 3 (6 × CMC)

**Table 2:** Growth rates of different benthic foraminifera species when exposed to additions of Cu, Cd, Zn, and Hg. Criteria maximum concentrations (CMC) were calculated based on the source published data according to the screening quick reference tables (SQuiRTs) of National Oceanic and Atmospheric Administration (NOAA) [41]. Growth rates were modified based on the source published data.

Species	HMs	Concentration Relative to CMC	Modified Growth Rates [Chamber Week^−1^]	Time [Day]	Source
*Ammonia tepida*	Cu + Mn + Ni	Control	0.10	82	Modified after [21]
		5 fold 13 × CMC 3 µg L^−1^ 0.1 × CMC	0.07		
		10 fold 22 × CMC 6 µg L^−1^ 0.3 × CMC	0.05		
		20 fold 44 × CMC 11 µg L^−1^ 0.5 × CMC	0.03		
*Amminia tepida*	Cd	Control 25 × CMC 63 × CMC 125 × CMC 250 × CMC 500 × CMC	0.20 ± 0.02 0.23 ± 0.01 0.20 ± 0.02 0.16 ± 0.02 0.10 ± 0.02 0 ± 0	30	Modified after [22]
*Pseudotriloculina rotunda*	Zn	Control 0.1 × CMC 1 × CMC 11 × CMC 111 × CMC 1111 × CMC	0.17 ± 0.02 0.16 ± 0.04 0.08 ± 0.01 0.04 ± 0.04 0.07 ± 0.02 0 ± 0	70	Modified after [24]
*Rosalina leei*	Hg	Control 0.01 × CMC 0.02 × CMC 0.03 × CMC 0.04 × CMC 0.06 × CMC 0.07 × CMC 0.08 × CMC 0.09 × CMC 0.10 × CMC	**[µm day^−1^]** 2.3 1.4 0.8 1.2 1.0 0.5 0.3 0.5 0.7 0.3	66	Modified after [20]

## Supplementary Materials

The following are available online at https://www.mdpi.com/1660-4601/17/10/3741/s1, Supplementary Material 1: Solution validation and conditions monitoring, Supplementary Material 2: Results and statistics.
