# Supplementary material for: Shell Growth of Large Benthic Foraminifera under Heavy Metals Pollution: Implications for Geochemical Monitoring of Coastal Environments
Source: Int J Environ Res Public Health. 2020 May 25;17(10):3741. doi: 10.3390/ijerph17103741 (PMC7277671; doi:10.3390/ijerph17103741)
Supplement: Supplementary file 1 [file ijerph-17-03741-s001.zip › Supplementary Material 1.pdf]

Shell growth of large benthic foraminifera under heavy metals pollution: Implications for geochemical monitoring of coastal environments

Nir Ben-Eliahu[1], Barak Herut[2], Eyal Rahav[2], Sigal Abramovich[1]

[1]Geological and Environmental Sciences Ben Gurion University of the Negev, Beer Sheva 84105, Israel

[2]Israel Oceanographic & Limnological Research, Haifa 31080, Israel

Supplementary material 1: Solution validation and conditions monitoring

Solutions preparation

20 liters of seawater were collected from Tel Shikmona station at a water depth of ~1 meter into 2 high-density polyethylene (HDPE) containers of 10 liters. The seawater was filtered using a peristaltic pump and 0.45 micro-meter filters (Cellulose Nitrate, Whatman) at the Israel Oceanographic and Limnological Research (IOLR). Stock solutions of Cd, Cu and Pb were prepared using the HM salts: $CdCl_2$, $C_4H_6O_4Pb \times 3H_2O$ (Sigma-Aldrich, 99.99% trace metals basis) and $CuCl_2$ (Sigma-Aldrich, 99.999% trace metals basis) and miliQ water inside LDPE bottles. Solutions were prepared weekly from the stock solutions and the filtered seawater. All of the sampling and experimental materials (LDPE pipettes, polypropylene tubes, LDPE bottles, HDPE bottles) were pre-cleaned by washing 3 times with miliQ water, soaking with 10% HCl for 24 hours (Analytical grade) and washing 3 times.

To validate the stability of the solutions over a 1-week period time, samples of the weekly made solutions were kept at pH <2 by adding ultrapure nitric acid (HNO_3) (J. T. Baker) until further analyzed for Cd, Cu and Pb concentrations. The samples were analyzed using an inductively coupled plasma-mass spectrometer (ICP-MS; Agilent 7500cx) at the Institute of Earth Sciences, the Hebrew University of Jerusalem. Standard reference samples (U.S Geological Survey Standards: T-229; T-235) were examined after calibration and at the end of the analysis for accuracy assessment.

Culturing conditions monitoring

pH measurements of *A. lobifera* and *S. orbiculus* (Supplementary Figure 1) were performed only on one replicate measured after the preparation of the solutions. The solutions were prepared weekly for six weeks with pH values ranging between 8.19-8.07±0.01. After every week of culturing, the pH of the solution was remeasured in one replicate of every specie. The lowest pH value measured in *A. lobifera* is 7.8, 7.9, 7.9 and 7.6 for the control, Cd, Cu and Pb treatments accordingly. In the *S. orbiculus* treatment, the lowest value measured was 7.9 in the Pb treatment. Among these results,

pH value of 7.6 found in the Pb treatment of *A. lobifera* is known to reduce the growth rates of several benthic foraminifera species yet, the growth rates results in this study show that the Pb treated *A. lobifera* specimens had similar growth rates to the control and were not affected, therefore the pH level probably did not have a negative effect on shell growth.

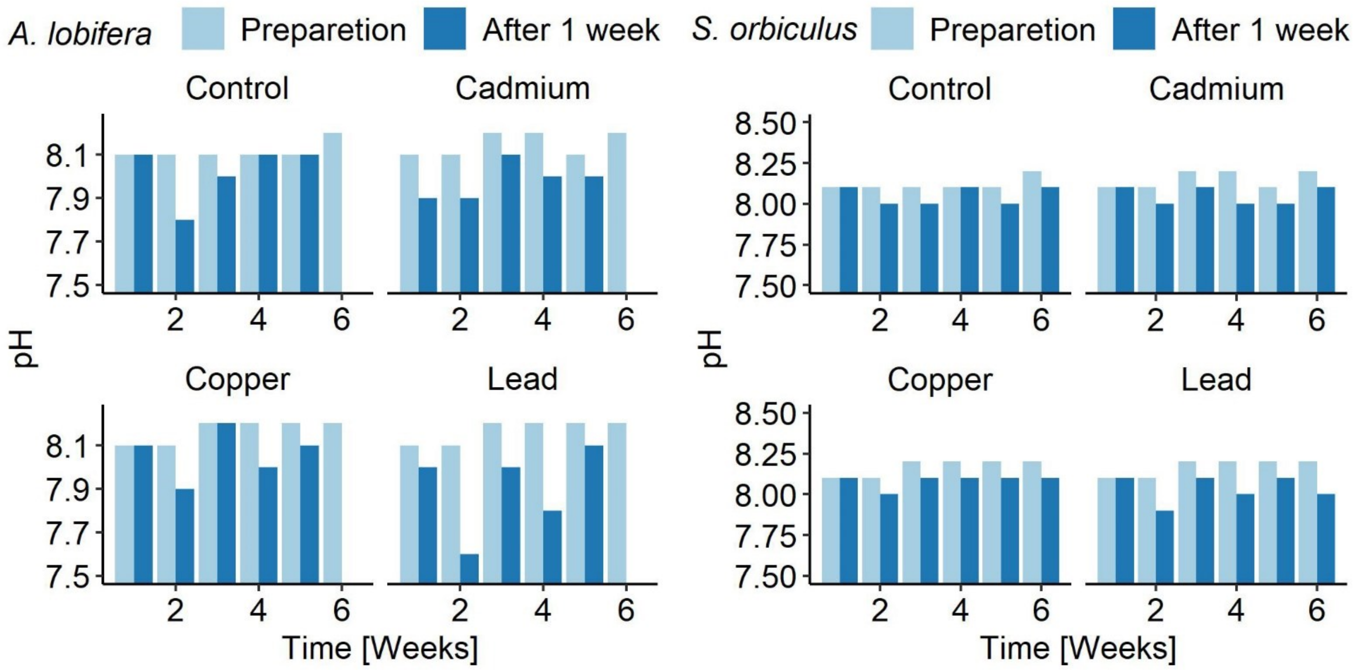

Supplementary Figure 2: pH measurements of *A. lobifera* and *S. orbiculus*.

pH measurements of the solutions experiment of *A. lessonii* (Supplementary Figure 3) were done on all three replicates including a blank solution without specimens. The pH was measured after preparation of the solutions weekly for 4 weeks with pH values ranging between 8.26-8.20±0.01. After every week of culturing, the pH of the solution was remeasured in all three replicates including. Measurements of the blank solutions after preparation and after one week have similar values ranging from 8.0-8.3. The lowest pH value measured in *A. lessonii* is 8.0, 7.8, 8, and 7.8 for the control, Cd, Cu and Pb treatments accordingly, values which are not thought to affect the specimens. Among the control, replicate two has a lower pH value of 7.6 after one week of culturing. This value is known to reduce the growth rates of several benthic foraminifera species yet, the growth rates between the control replicates are similar, therefore, the pH level probably did not have a negative effect on shell growth.

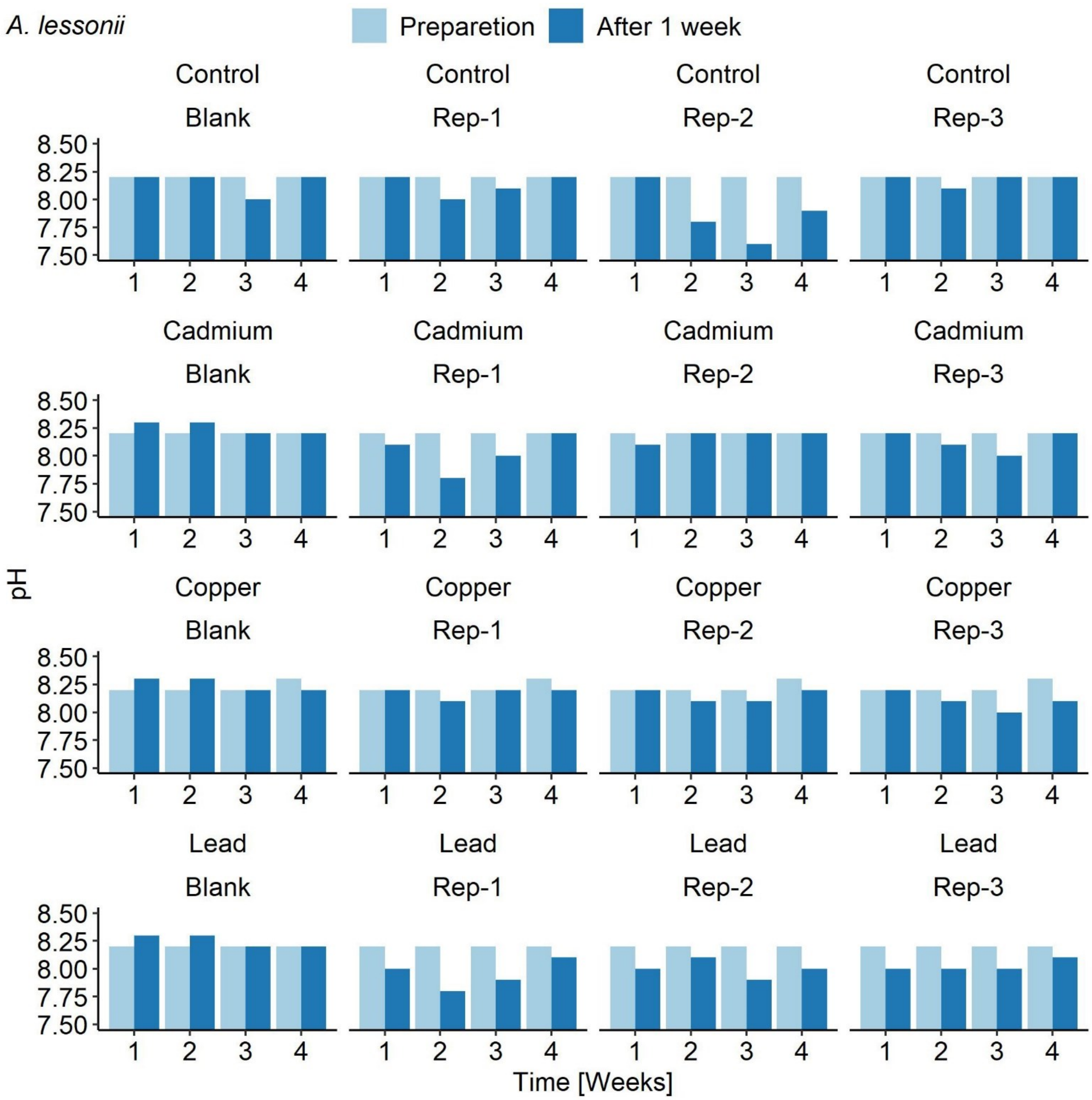

Supplementary Figure 4: pH measurements of the solutions experiment of *A. lessonii*.

Salinity measurements of the solutions experiments of *A. lessonii* (Supplementary Figure 5) were done on all three replicates including a blank solution without specimens. Note: the lower salinity value in the blank of the Pb treatment after 1 week, probably the result of a measurement error. The salinity was stable in all treatments and replicates, ranging between 38-39 ppt, values which are not known to cause negative effects on the growth of foraminifera. The salinity result of the blank replicate in the Pb treatment after 1 week has a relatively lower value of 38 ppt, probably the result of a measurement error.

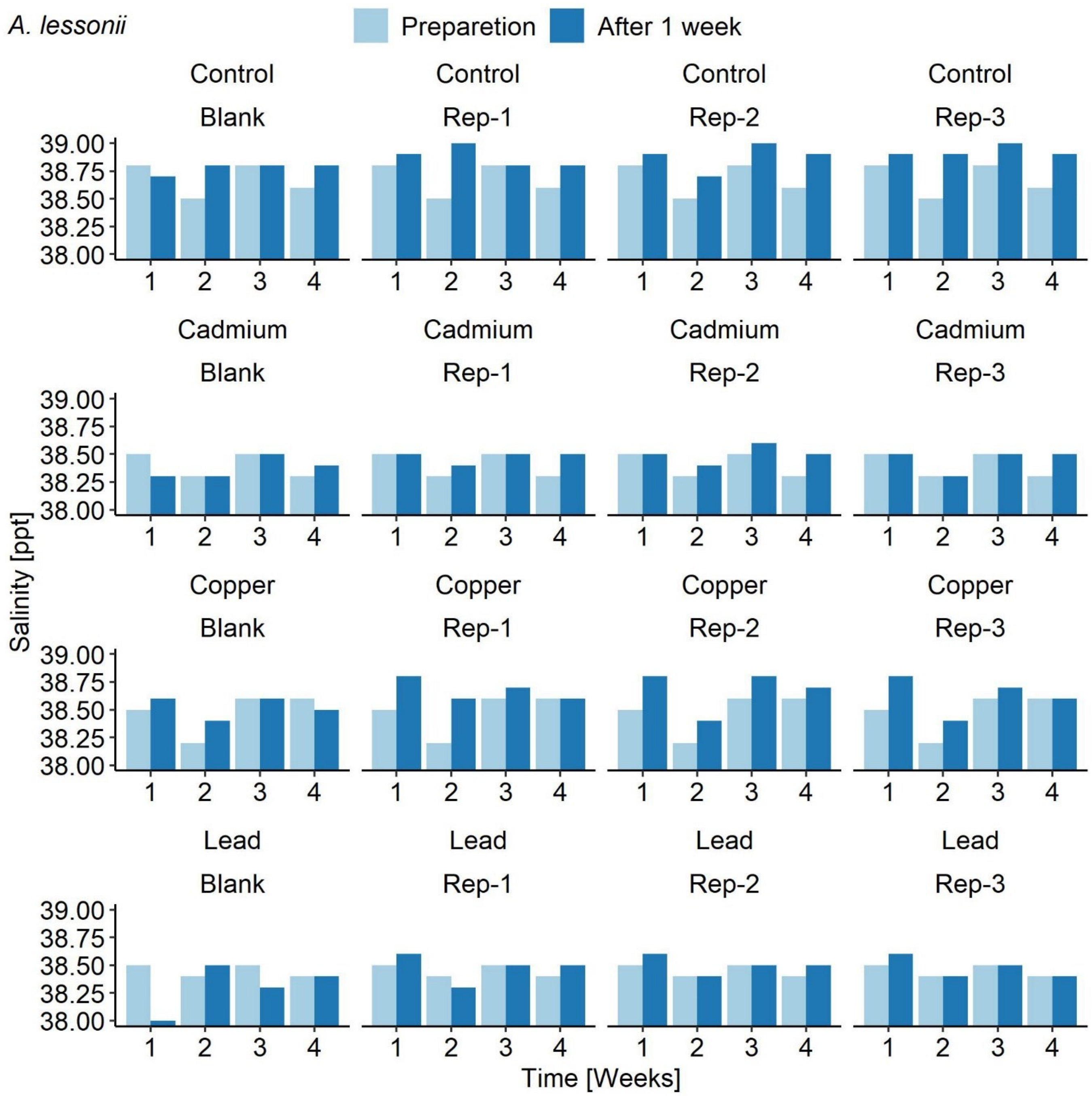

Supplementary Figure 6: Salinity measurements of the solutions experiments of *A. lessonii*.
