# Supplementary material for: Shell Growth of Large Benthic Foraminifera under Heavy Metals Pollution: Implications for Geochemical Monitoring of Coastal Environments
Source: Int J Environ Res Public Health. 2020 May 25;17(10):3741. doi: 10.3390/ijerph17103741 (PMC7277671; doi:10.3390/ijerph17103741)
Supplement: Supplementary file 1 [file ijerph-17-03741-s001.zip › Supplementary Material 2.pdf]

Shell growth of large benthic foraminifera under heavy metals pollution: Implications for geochemical monitoring of coastal environments

Nir Ben-Eliahu[1], Barak Herut[2], Eyal Rahav[2], Sigal Abramovich[1]

[1]Geological and Environmental Sciences Ben Gurion University of the Negev, Beer Sheva 84105, Israel

[2]Israel Oceanographic & Limnological Research, Haifa 31080, Israel

Supplementary material 2: Results and statistics

All statistics were performed using R software version 3.5.3. Most of the data was found to have non-normal distribution according to the Shapiro-Wilk test, so the Mann-Whitney nonparametric test was used between the control and the HMs treatments. Significant differences were accepted at $P<0.05$.

Supplementary Table 1: Added new chambers and area differences of all specimens in the experiments. Error is the standard deviation of three replicates. *** Represents data that doesn't fit the required statistical test assumptions.

Specie	Time [day]	Treatment	Number of Specimens	New Chamber	Difference from the control
A. lobifera	31	Control	84	3.6±1.1	
		Cd	87	3.0±1.4	***Mann-Whitney test, P<0.05
		Cu	90	3.0±1.0	Mann-Whitney test, P<0.05
		Pb	90	3.6±1.2	
A. lessonii	24	Control	86	3.2±1.4	
		Cd	88	3.1±1.6	
		Cu	89	3.1±1.6	
		Pb	89	3.4±1.7	
S. orbiculus	32	Control	49	2.5±0.8	
		Cd	49	2.0±0.9	Mann-Whitney test, P<0.05
		Cu	51	1.8±0.7	Mann-Whitney test, P<0.05
		Pb	49	1.7±0.8	***Mann-Whitney test, P<0.05
				Area Addition [mm^2]	
		Control	49	318±169	
		Cd	49	247±161	
		Cu	51	273±158	
		Pb	49	230±157	

Supplementary Table 2: F test (compare two variances) and Shapiro-Wilk test (normality) to compare newly added chambers between two independent treatments of *A. lobifera*. Statistical differences accepted at $P<0.05$ are mark in bold.

Groups	F test to compare two variances			Shapiro-Wilk normality test	
	F	df	p-value	W	p-value
Control-Cd	1.803	86	**0.007454**	0.94453	**3.189e-06**
Control-Cu	0.84099	89	0.422	0.91716	**2.273e-08**
Control-Pb	1.3156	89	0.2074	0.92755	**1.222e-07**

Supplementary Table 3: Mann-Whitney test to compare newly added chambers between two independent treatments of *A. lobifera*. Statistical differences accepted at P<0.05 are mark in bold.

Groups	Mann-Whitney test	
	W	p-value
Control-Cd	2649.5	**0.001388**
Control-Cu	2158	**3.695e-07**
Control-Pb	3801	0.9487

Supplementary Table 4: F test (compare two variances) and Shapiro-Wilk test (normality) to compare newly added chambers between two independent treatments of *A. lessonii*. Statistical differences accepted at P<0.05 are mark in bold.

Groups	F test to compare two variances			Shapiro-Wilk normality test	
	F	df	p-value	W	p-value
Control-Cd	1.3873	87	0.1315	0.91347	**1.29e-08**
Control-Cu	0.80185	85	0.3073	0.91818	**2.477e-08**
Control-Pb	0.63825	85	**0.03852**	0.94122	**1.32e-06**

Supplementary Table 5: Mann-Whitney test to compare newly added chambers between two independent treatments of *A. lessonii*. Statistical differences accepted at P<0.05 are mark in bold.

Groups	Mann-Whitney test	
	W	p-value
Control-Cd	3721.5	0.8475
Control-Cu	4040	0.5142
Control-Pb	3549	0.3962

Supplementary Table 6: F test (compare two variances) and Shapiro-Wilk test (normality) to compare newly added chambers between two independent treatments of *S. orbiculus*. Statistical differences accepted at P<0.05 are mark in bold.

Groups	F test to compare two variances			Shapiro-Wilk normality test	
	F	df	p-value	W	p-value
Control-Cd	1.238	48	0.4624	0.87364	**1.242e-07**
Control-Cu	1.1979	48	0.5283	0.8597	**2.753e-08**

Control-Pb	1.1351	48	0.6625	0.87775	**1.833e-07**

Supplementary Table 7: Mann-Whitney test to compare newly added chambers between two independent treatments of *S. orbiculus*. Statistical differences accepted at P<0.05 are mark in bold.

Groups	Mann-Whitney test	
	W	p-value
Control-Cd	804	**0.002808**
Control-Cu	1783.5	**8.244e-05**
Control-Pb	1798	**4.214e-06**

Supplementary Table 8: F test (compare two variances) and Shapiro-Wilk test (normality) to compare area differences between two independent treatments of *S. orbiculus*. Statistical differences accepted at P<0.05 are mark in bold.

Groups	F test to compare two variances			Shapiro-Wilk normality test	
	F	df	p-value	W	p-value
Control-Cd	0.90011	48	0.7169	0.91525	**9.607e-06**
Control-Cu	1.1496	48	0.626	0.91276	**5.933e-06**
Control-Pb	1.1587	48	0.6119	0.92094	**1.897e-05**

Supplementary Table 9: Mann-Whitney test to compare area differences between two independent treatments of *S. orbiculus*. Statistical differences accepted at P<0.05 are mark in bold.

Groups	Mann-Whitney test	
	W	p-value
Control-Cd	863.5	**0.0168**
Control-Cu	1484	0.1066
Control-Pb	1641,	**0.00177**

Supplementary Table 10: Average Chl *a* concentrations normalized to the area of the specimens. Error is the standard deviation of the three replicates.

Specie	Treatment	Number of Specimen	Chl *a* [ng mm^{-2}]	Difference from the control
A. lobifera	Control	12	0.11±0.03	
	Cd	12	0.14±0.07	
	Cu	12	0.14±0.03	
	Pb	12	0.11±0.03	
A. lessonii	Control	18	0.05±0.02	
	Cd	18	0.05±0.02	
	Cu	18	0.06±0.02	Mann-Whitney test, P<0.05
	Pb	18	0.03±0.02	Mann-Whitney test, P<0.05
S. orbiculus	Control	11	0.09±0.02	
	Cd	11	0.09±0.02	
	Cu	12	0.09±0.02	
	Pb	11	0.11±0.02	Mann-Whitney test, P<0.05

Supplementary Table 11: F test (compare two variances) and Shapiro-Wilk test (normality) to compare Chl *a* concentrations between two independent treatments of *A. lobifera*. Statistical differences accepted at P<0.05 are mark in bold.

Groups	F test to compare two variances			Shapiro-Wilk normality test	
	F	df	p-value	W	p-value
Control-Cd	3.5339	11	**0.04709**	0.82022	**0.0006426**
Control-Cu	1.0785	11	0.9025	0.95787	0.2168
Control-Pb	1.0421	11	0.9467	0.94557	0.2168

Supplementary Table 12: Mann-Whitney test to compare Chl *a* concentrations between two independent treatments of *A. lobifera*. Statistical differences accepted at P<0.05 are mark in bold.

Groups	Mann-Whitney test	
	W	p-value
Control-Cd	84,	0.5065
Control-Cu	42.5	0.09335
Control-Pb	85	0.4703

Supplementary Table 13: F test (compare two variances) and Shapiro-Wilk test (normality) to compare Chl *a* concentrations between two independent treatments of *A. lessonii*. Statistical differences accepted at P<0.05 are mark in bold.

Groups	F test to compare two variances			Shapiro-Wilk normality test	
	F	df	p-value	W	p-value
Control-Cd	0.4498	17	0.109	0.96986	0.4218
Control-Cu	2.3042	17	0.09444	0.98986	0.9813
Control-Pb	2.0343	17	0.1532	0.94823	0.09198

Supplementary Table 14: Mann-Whitney test to compare Chl *a* concentrations between two independent treatments of *A. lessonii*. Statistical differences accepted at P<0.05 are mark in bold.

Groups	Mann-Whitney test	
	W	p-value
Control-Cd	166.5	0.8992
Control-Cu	97.5	**0.04279**
Control-Pb	268	**0.0008384**

Supplementary Table 15: F test (compare two variances) and Shapiro-Wilk test (normality) to compare Chl *a* concentrations between two independent treatments of *S. orbiculus*. Statistical differences accepted at P<0.05 are mark in bold.

Groups	F test to compare two variances			Shapiro-Wilk normality test	
	F	df	p-value	W	p-value
Control-Cd	0.91866	10	0.8959	0.916	0.06285
Control-Cu	1.5477	10	0.484	0.94428	0.2219
Control-Pb	1.1184	10	0.8631	0.94185	0.2161

Supplementary Table 16: Mann-Whitney test to compare Chl *a* concentrations between two independent treatments of *S. orbiculus*. Statistical differences accepted at P<0.05 are mark in bold.

Groups	Mann-Whitney test	
	W	p-value
Control-Cd	51.5	0.5761
Control-Cu	64	0.9264
Control-Pb	28.5	**0.03843**

Supplementary Table 17: Growth rate results calculated by the area differences in *S. orbiculus* and the number of new chambers added, in *A. lobifera*, *A. lessonii* and *S. orbiculus*. Error is the standard deviation of the three replicates.

Specie	Time [day]	Treatment	Growth [Chamber Week^{-1}]
A. lobifera	31	Control	0.8±0.3
		Cd	0.7±0.3
		Cu	0.7±0.2
		Pb	0.8±0.3
A. lessonii	24	Control	0.9±0.4
		Cd	0.9±0.5
		Cu	0.9±0.5
		Pb	1.0±0.5
S. orbiculus	32	Control	0.5±0.2
		Cd	0.4±0.2
		Cu	0.4±0.2
		Pb	0.3±0.2
		Growth Rate [Area % Day^{-1}]	
		Control	2.1±0.7
		Cd	1.6±0.6
		Cu	1.8±0.5
		Pb	1.5±0.7

Supplementary Table 18: Two-way ANOVA test comparing relative growth rates variances between different treatments of Cd and Cu at different CMC additions. Statistical differences accepted at P<0.05 are mark in bold.

	df	Sum Sq	Mean Sq	F value	p-value
CMC	1	8855.3	8855.3	90.787	**1.869e-08**
Treatment	1	1732.5	1732.5	17.763	**0.0005211**
CMC:Treatment	1	3840.8	3840.8	39.377	**6.447e-06**
Residuals	18	1755.7	97.5		

Supplementary Table 19: Number of specimens used in the Image analyses. Dead specimens were excluded from the analyses.

Treatment	Replicate	Number of specimens (Replicate)	Number of specimens (Treatment)
Control	Rep-1	27/29	81/86
	Rep-2	25/28	
	Rep-3	29/29	
Cd	Rep-1	23/30	82/88
	Rep-2	30/30	
	Rep-3	29/28	
Cu	Rep-1	28/29	82/87
	Rep-2	27/29	
	Rep-3	27/29	
Pb	Rep-1	27/29	84/89
	Rep-2	29/30	
	Rep-3	28/30	

Supplementary Table 20: Average I_{red} values. For imaging settings refer to S1. I_{red} error of the replicates is the standard deviation of specimens I_{red} values. Treatment error is the standard deviation of three replicates.

Treatment	Replicate	Average I_{red} [DN s^{-1}] Replicate	Average I_{red} [DN s^{-1}] Treatment	Difference from the Control
Control	Rep-1	386±75	359±53	
	Rep-2	332±26		
	Rep-3	358±30		
Cd	Rep-1	340±32	331±32	
	Rep-2	322±31		
	Rep-3	333±32		
Cu	Rep-1	303±34	323±31	Mann-Whitney P<0.05
	Rep-2	323±14		
	Rep-3	344±26		
Pb	Rep-1	301±29	293±26	Mann-Whitney P<0.05
	Rep-2	294±20		
	Rep-3	284±25		

Supplementary Table 21: F test (compare two variances) and Shapiro-Wilk test (normality) to compare I_{red} values between two independent treatments of *A. lessonii*. Statistical differences accepted at P<0.05 are mark in bold.

Groups	F test to compare two variances			Shapiro-Wilk normality test	
	F	df	p-value	W	p-value
Control-Cd	1.0429	81	0.8506	0.92116	**8.859e-05**
Control-Cu	1.4751	81	0.07991	0.95392	**0.005068**
Control-Pb	1.4751	81	0.07991	0.89376	**4.225e-06**
Cd-Pb	1.5383	81	0.0525	0.93519	**7.81e-07**
Cu-Pb	1.4751	81	0.07991	0.97158	**0.001727**
Cd-Cu	1.0429	81	0.8506	0.97294	**0.002662**

Supplementary Table 22: Mann-Whitney test to compare I_{red} values between two independent treatments of *A. lessonii*. Statistical differences accepted at P<0.05 are mark in bold.

Groups	Mann-Whitney test	
	W	p-value
Control-Cd	3725	0.2295
Control-Cu	5522.5	**1.629e-11**
Control-Pb	5522.5	**1.629e-11**
Cd-Pb	5707	**1.006e-13**
Cu-Pb	5522.5	**1.629e-11**
Cd-Cu	3725	0.2295

Supplementary Table 23: Survival rates. Living, dead, lost and specimens which overwent reproduction event. Specimens over going reproduction were considered as living specimens. Survival rate error is the standard deviation of three replicates.

Specie	Treatment	Replicate	Living Specimens	Dead Specimens	Lost Specimens	Reproduction	Survival rate [%]
A. lobifera	Control	Rep-1	30	0	0	0	98±2
		Rep-2	24	1	5	0	
		Rep-3	29	1	0	0	
	Cd	Rep-1	24	3	3	0	94±6
		Rep-2	30	0	0	0	
		Rep-3	28	2	0	0	
	Cu	Rep-1	28	2	0	0	93±4
		Rep-2	27	3	0	0	
		Rep-3	29	1	0	0	
	Pb	Rep-1	30	0	0	0	97±6
		Rep-2	27	3	0	0	
		Rep-3	30	0	0	0	
A. lessonii	Control	Rep-1	27	1	1	1	96±2
		Rep-2	24	2	2	2	
		Rep-3	28	1	1	0	
	Cd	Rep-1	24	2	2	2	97±4
		Rep-2	30	0	0	0	
		Rep-3	28	1	0	1	
	Cu	Rep-1	28	2	0	0	90±3
		Rep-2	26	4	0	0	
		Rep-3	27	3	0	0	
	Pb	Rep-1	25	4	0	1	96±8
		Rep-2	29	0	0	1	
		Rep-3	28	0	1	1	
S. orbiculus	Control	Rep-1	17	0	0	0	100±0
		Rep-2	15	0	0	0	
		Rep-3	9	0	8	0	
	Cd	Rep-1	14	2	1	0	92±3
		Rep-2	16	1	0	0	
		Rep-3	16	1	0	0	
	Cu	Rep-1	16	1	0	0	94±6
		Rep-2	15	2	0	0	
		Rep-3	17	0	0	0	
	Pb	Rep-1	16	1	0	0	92±3
		Rep-2	14	2	1	0	
		Rep-3	16	1	0	0	
